# Over-expression of BMPR-IB reduces the malignancy of glioblastoma cells by upregulation of p21 and p27Kip1

**DOI:** 10.1186/1756-9966-31-52

**Published:** 2012-05-31

**Authors:** Shuang Liu, Feng Yin, Wenhong Fan, Shuwei Wang, Xin-ru Guo, Jian-ning Zhang, Zeng-min Tian, Ming Fan

**Affiliations:** 1 Department of Neurosurgery, Navy General Hospital, 100048, Beijing, China; 2 Department of Brain Protection & Plasticity Research, Beijing Institute of Basic Medical Sciences, Taiping Road 27, Beijing, 100850, People’s Republic of China; 3 Beijing Institute Neuroscience, Capital Medical University, Beijing, 100069, China

**Keywords:** 1. BMPR-IB, 2. Glioblastoma, 3. Growth inhibition, 4. Differentiation

## Abstract

**Background:**

In our previous study, we detected decreased expression of phospho-Smad1/5/8 and its upstream signaling molecule, bone morphogenetic protein receptor IB subunit (BMPR-IB), in certain glioblastoma tissues, unlike normal brain tissues. In order to clarify the functional roles and mechanism of BMPR-IB in the development of glioblastoma, we studied the effects of BMPR-IB overexpression on glioblastoma cell lines in vitro and in vivo.

**Methods:**

We selected glioblastoma cell lines U251, U87, SF763, which have different expression of BMPR-IB to be the research subjects. Colony formation analysis and FACS were used to detect the effects of BMPR-IB on the growth and proliferation of glioblastoma cells in vivo. Immunofluresence was used to detect the differentiation changes after BMPR-IB overexpression or knocking-down. Then we used subcutaneous and intracranial tumor models to study the effect of BMPR-IB on the growth and differentiation of glioblastoma cells in vivo. The genetic alterations involved in this process were examined by real-time PCR and western blot analysis.ed.

**Results and conclusion:**

Forced BMPR-IB expression in malignant human glioma cells, which exhibit lower expression of BMPR-IB, induced the phosphorylation and nuclear localization of smad1/5/8 and arrested the cell cycle in G1. Additionally, BMPR-IB overexpression could suppress anchorage-independent growth and promote differentiation of theses glioblastoma cells. Furthermore, overexpression of BMPR-IB inhibited the growth of subcutaneous and intracranial tumor xenografts and prolonged the survival of mice injected intracranially with BMPR-IB-overexpressing glioblastoma cells. Conversely, inhibition of BMPR-IB caused SF763 malignant glioma cells, a line known to exhibit high BMPR-IB expression that does not form tumors when used for xenografts, to show increased growth and regain tumorigenicity in a nude mouse model system, ultimately shortening the survival of these mice. We also observed significant accumulation of p21 and p27kip1 proteins in response to BMPR-IB overexpression. Our study suggests that overexpression of BMPR-IB may arrest and induce the differentiation of glioblastoma cells due to upregulation of p21 and p27kip1 in vitro and that in vivo and decreased expression of BMPR-IB in human glioblastoma cells contributes to glioma tumorigenicity. BMPR-IB could represent a new potential therapeutic target for malignant human gliomas.

## Background

Malignant gliomas are the most common primary tumors in the brain; they are destructive, invasive, and the most highly vascularized lethal tumors observed in humans. Gliomas are classified into grades I – IV according to their histological degree of malignancy by the WHO criterion. Despite recent progress in combination therapies, the median survival of patients with glioblastoma (WHO grade IV) is less than 14–15 months [1]. Advances in the treatment of malignant gliomas will require improved understanding of the biology and molecular mechanisms of glioma development and progression. Many studies show that the malignant transformation of glioma is a consequence of the stepwise accumulation of genetic alterations that lead to aberrant regulation of proliferation and differentiation signals and disruption of the apoptotic pathway [1]. Recent research on the molecular basis of gliomas and the implications for targeted therapeutics has focused on the PTEN, EGFR and VEGF signaling pathways [2-4]. These pathways, while important, represent only a subset of the multiple molecular mediators of glioma tumorigenesis and malignancy [1].

A previous study by our group showed that the expression of bone morphogenetic protein receptor IB subunit (BMPR-IB) is decreased in most malignant human glioma tissues, including anaplastic astrocytomas and glioblastomas. Furthermore, the low expression of BMPR-IB was found to contribute to a lower ratio of phospho-Smad1/5/8 to Smad1/5/8 expression, which correlates significantly with poor patient survival [5]. Thus, it would not be unreasonable to speculate that BMP signals may participate in the development and progression of gliomas.

BMPs are the subclass of the transforming growth factor-β (TGF-β) superfamily, including more than 20 members. BMP ligands and receptor subunits are present throughout neural development and mediate a diverse array of developmental processes, including cellular survival, proliferation, morphogenesis, lineage commitment, differentiation and apoptosis of neural stem cells in the CNS [6-8]. Additionally, during regional and cellular maturation, BMPs can mediate long-range signaling by acting as gradient morphogens, or they can mediate short-range signaling by modulating cell-cell communication [6,7,9]. BMP signals transduce intracellular signals through type I (BMP-RIA and BMP-RIB) and type II (BMP-RII) serine/threonine kinase receptors. Binding of BMPs to BMPR-II results in phosphorylation of BMPR-I and downstream Smad proteins. BMPs activate Smad1/5/8, which can associate with Smad4 in a heterodimeric complex upon phosphorylation that is translocated to the nucleus, where it activates transcription [10-13].

Although the BMP pathways have emerged as important contributors to many human neoplastic conditions [14,15], the role of BMPs/BMPRs in human glioma has not been completely defined. In the present study, we continued to investigate how BMPR-IB regulates the growth of glioblastomas.

## Methods

### Cell lines and cell culture

The human malignant glioma cell lines SF126, SF763, and M17 were obtained from the American Type Culture Collection. The glioblastoma cell line U-251 and normal human astrocytes, which were described previously **(5)**, were also used. These cell lines were cultured in D/F12 medium supplemented with 10% fetal bovine serum (FBS), (Hyclone USA).

### Animals

The athymic BALB/c nude mice (female), which weight from 25 to 28 g, were purchased from the Animal Center of the Chinese Academy of Medical Science. The mice were bred in laminar flow cabinets under specific pathogen-free conditions and handled according to the policies and standards of Laboratory Animal Care in China.

### Stable transfection of glioma cells

To generate a recombinant AAV serotype 2 –BMPR-IB (rAAV2-BMPR-IB) viral vector, full-length cDNA for human BMPR-IB was obtained by EcoRI and BamH1 digestion and subcloned into the pSNAV plasmid (Invitrogen) and was then recombined into rAAV2. U87 and U251MG cells were infected with AAV-BMPR-IB or control virus to generate BMPR-IB-overexpressing glioblastoma cells. To generate a BMPR-IB small interfering RNA (siRNA) expression vector for gene knockdown studies, four BMPR-IB siRNAs were designed and synthesized (Invitrogen, USA). A siRNA with the sequence 5′-GGACCCAGUUGUACCUAAUdTdT-3′ was determined to be the most effective siRNA for inhibiting BMPR-IB expression. The BMPR-IB siRNA was further incorporated into the pSilencer plasmid (Ambion, USA). SF763 cells were transfected with the BMPR-IB siRNA expression vector (si-BMPR-IB) or the control vector (si-control). The cell lines, which stably expressed BMPR-IB siRNA, were isolated by neomycin (G418) selection.

### Quantitative real-time RT-PCR

Total RNA, which derived from glioma cells, was prepared using TRIzol (Gibco), and further purified using the RNeasy Mini Kit (Qiagen). Real-time PCR was performed according to the manufacturer’s instructions using an ABI Prism 7900 sequence detection system (Applied Biosystems, USA). Primers and probes for p21, p27, p53, CDK2, CDK4, Skp2, BMPR-IB (human) and GAPDH were obtained from Applied Biosystems, USA. Additional file 1: Table S 1 shows the forward and reverse primer sequences of theses genes. All samples were tested in triplicate. The relative number of target transcripts was normalized to the number of human GAPDH transcripts in the same sample. The relative quantitation of target gene expression was performed using the standard curve or comparative cycle threshold (Ct) method.

### Western blot analysis

Whole-cell lysates were isolated from glioma cells and the transplanted glioma tissues **(5)**. Standard western blotting was performed with monoclonal antibodies against human BMPR-IB, p21, p27^KIP1^, Skp2, Cdk2, Cdk4, p53, GFAP, Nestin and β-actin proteins(Santa Cruz Biotechnology,USA) and the corresponding secondary antibodies (anti-rabbit IgG, anti-mouse IgG, and anti-goat IgG; Abcam, USA). Human β-actin was used as a loading control. These proteins were detected using the Amersham enhanced chemiluminescence system according to the manufacturer’s instructions.

### Immunofluorescent staining

At 48 h following AAV-BMPR-IB infection, the U251 and U87 cells were fixed in 4% paraformaldehyde-PBS. After incubation with 0.1% Triton-PBS for 30 min and blocking with 1% bovine serum albumin-PBS for 2 h in room temperature, the cells were then incubated with the primary antibodies overnight in 4°C at the concentration recommended by the supplier (a rabbit anti-phospho-Smad1/5/8 antibody (Cell signal), a goat anti-BMPR-IB antibody (Santa Cruz Biotechnology) and a mouse anti-GFAP antibody (Sigma)). After washing with 0.1% Triton-PBS three times, cells were incubated with RBITC-conjugated rabbit anti-goat IgG and FITC-conjugated goat anti-rabbit IgG (Santa Cruz Biotechnology) for 2 h in room temperature. The cell nuclei were stained with DAPI. The stained cells were visualized and mounted with a confocal laser scanning microscope (Olympus).

### Analysis of cell cycle distribution

Glioma cells were harveseted and washed with 1×PBS three times, then fixed by 70% Ethanol for 1 h. Ethanol-fixed cells were treated with RNase (10 mg/ml in PBS) and stained with propidium iodide (100 μg/mL in PBS) for 30 min at 37°C. Stained cells were tranfered to FACS tubes and detected using flow cytometry (Becton Dickinson Immunocytometry Systems, San Jose, CA).

### Colony formation in soft agar

U87 and U251 cells were infected with either rAAV2-BMPR-IB or the control vector rAAV2, SF763 cells were stably transfected with the BMPR-IB siRNA oligonucleotide or control siRNA. After 48 h of infection, cells were trypsinized, and 2×10^4^ cells were mixed with a 0.5% agar solution in DMEM/F12 containing 10% FBS and 200 μg/mL neomycin, then layered on top of 0.70% agar in 35 mm culture plates. The plates were incubated at 37°C in a humidified incubator for 10–14 days. Colonies were then stained with 0.005% Crystal violet for 1 h, and counted using a dissecting microscopically in 8 randomly chosen microscope fields. Only colonies containing >50 cells were scored.

### Subcutaneous tumor growth

To study the kinetics of glioma cells growth in vivo, glioma cells (3 × 10^6^ or 1 × 10^7^ cells in 50 μl of PBS) were injected s.c. into the right armpit of nude mice. The diameter of the resulting tumors was measured once every 5 days. The ability of tumor formation was determined by measurement of the diameters of subcutaneous tumors.

### Intracranial human glioma xenograft model

Glioma cells (1 × 10^6^/4μl) were grown in metrigel for 2 h, then implanted into the right striatum of nude mice by stereotactic injection (0.2 μl/min). The injection coordinates were: anteroposterior = 0; mediolateral = 3.0 mm; and dorsoventral = 4.0 mm. Animals showing general or local symptoms were killed; the remaining animals were killed 90 days after glioma cell injection by perfusion of 4% formaldehyde. The brain of each mouse was harvested, fixed in 4% formaldehyde, and embedded in paraffin. Tumor formation and phenotype were determined by histological analysis of hematoxylin and eosin (H&E)-stained sections. Two independent experiments were performed, with five mice per group in each experiment.

### Histology and immunohistochemistry of xenograft tumors

Fixed Brain tissue specimens were embedded in paraffin, sectioned, and stained with H&E according to standard protocols. Tissue sections were immunostained using mouse anti- GFAP and goat anti-CD34 monoclonal antibodies (Santa Cruz Biotechnology,USA) to detect the growth, differentiation and angiogenesis of the xenografts.

### Statistics

All of the values were calculated as mean±SE. Student’s *t*-test was used to analysis the significance of the results in vitro, whereas the significance of the results in vivo was determined by the Mann–Whitney *U* test. Kaplan–Meier survival analysis was used to analysis the overall survival times of the glioblastoma nude mouse.

## Results

### Expression of members of the BMPs/Smad1/5/8 signaling pathway in different malignant glioma cell lines

**Figure 1:**
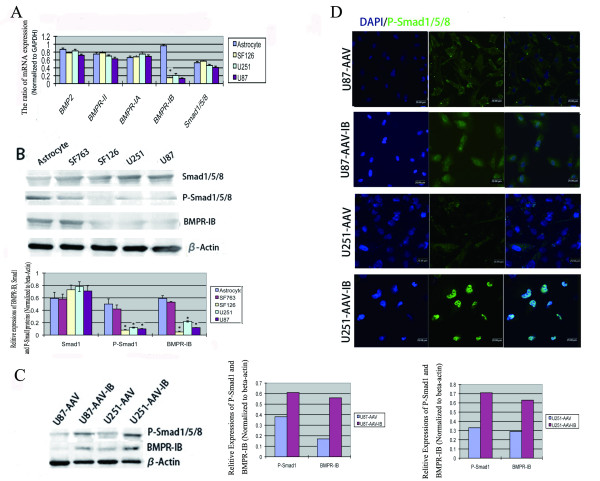
**Determination of BMPR-IB expression in normal human astrocytes and glioma cell lines.** (**A**) Real-time-RT-PCR was used to determine the mRNA expressions of BMPR-IB and other factors involved in BMP/BMPR signaling pathway. (**B**) Western blot analyses were employed to show the protein expression of BMPR-IB, P-Smad1/5/8 and Smad1/5/8 in glioblastoma cell lines(up). Statistical analysis of results from WB analysis(down). (**C**) Alterations in the expression of BMPR-IB and P-Smad1/5/8 after 48 h of BMPR-IB overexpression, determined by WB analysis. (**D**) Immunofluorescence analysis of the activation of Smad1/5/8 after 48 h of BMPR-IB infection.

We examined the mRNA and protein expressions of BMP2, BMPR-II, BMPR-IA, BMPR-IB and Smad1/5/8 in normal astrocytes and malignant glioma cell lines using real-time RT-PCR and western blot analysis, respectively. We found that the mRNA expression of BMPR-IB mRNA in all glioblastoma cell lines decreased compared to normal astrocytes, while the expression of the other genes remained similar between normal astrocytes and malignant glioma cell lines (Figure 1A). Furthermore, the protein expression of BMPR-IB and phospho-Smad1/5/8 in all malignant glioma cell lines was lower than the levels in normal astrocytes; intracellular protein expression of BMPR-IB was moderately lower in SF763 cells and drastically lower in other malignant glioma cell lines compared to normal astrocytes (Figure 1B). We overexpressed BMPR-IB in U87 and U251 cells following rAAV infection. Forty-eight hours after infection, a significant increase of BMPR-IB and phospho-smad1/5/8 protein expression was confirmed in the rAAV-BMPR-IB-infected U87 and U251 cell lines by western blot analysis (Figure 1C). Furthermore, immunofluorescent staining with an anti-phospho-smad1/5/8-specific antibody showed nuclear translocation of phospho-smad1/5/8 after 48 h of AAV-BMPR-IB infection (Figure 1D).

### Effects of BMPR-IB overexpression and knock-down on the cell cycle progression of glioblastoma cells

**Figure 2:**
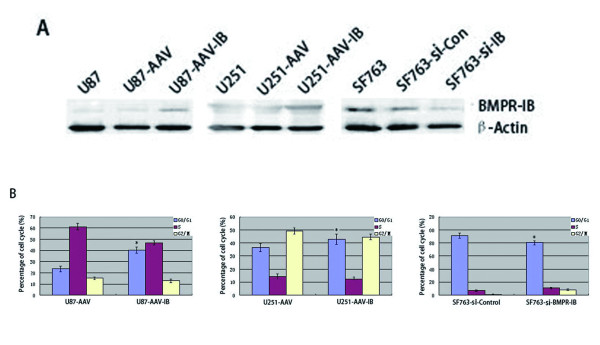
**Effects of altered BMPR-IB expression on the cell cycle of human glioblastoma cells.** (**A**) Western blot analysis of BMPR-IB expression in parental glioma cells, control vector–AAV and AAV-BMPR-IB-infected cells. (**B**) Cell cycle distribution analysis histogram. (Values are expressed as the mean±SD, n = 3. *, P < 0.05).

We overexpressed BMPR-IB with rAAV in U87 and U251 cells and suppressed BMPR-IB expression in SF763 cells with siBMPR-IB. Forty-eight hours after infection and transfection, a significant increase in BMPR-IB protein expression in the rAAV-BMPR-IB-infected U87 and U251 cell lines and a decrease in BMPR-IB protein expression in the BMPR-IB siRNA-transfected SF763 cell line were confirmed by western blot analysis (Figure 2A). Defects in the regulation of cell cycle progression are thought to be among the most common features of glioblastoma multiforme [1]. Therefore, we used flow cytometry to assess whether BMPR-IB expression could affect the cell cycle progression of glioblastoma cells. As shown in Figure 2B, the percentage of BMPR-IB-infected U87 and U251 cells in G1/G0 phase was higher compared to that of control vector rAAV-infected cells. Conversely, the percentage of si-BMPR-IB transfected SF763 cells in G0/G1 phase was lower relative to that of si-control-transfected SF763 cells.

### Effects of BMPR-IB overexpression and knock-down on the growth of glioblastoma cells in vitro

**Figure 3:**
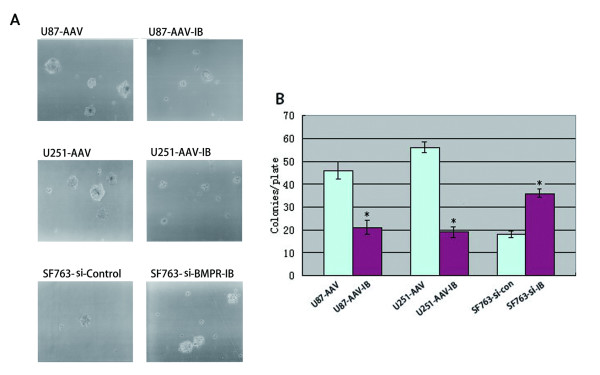
**Determination of anchorage-independent growth of human glioma cells with altered BMPR-IB expression using a soft-agar colony formation assay.** (**A**) Microphotographs of colonies. (**B**) Columns, the mean of the colony numbers on triplicate plates from a representative experiment (conducted twice); bars, SD. *, P < 0.001, as determined using Student’s *t*-test.

After 5 days of BMPR-IB overexpression or knock-down, the anchorage-independent growth of BMPR-IB-overexpressing glioblastoma cells was drastically inhibited, as shown by a decrease in the number and volume of colonies on soft agar compared with control cells, and the anchorage-independent growth of SF763 cells treated with siBMPR-IB was 2 times as high as that of the si-control-treated cells. BMPR-IB overexpression decreased the colony numbers of U251 and U87 by 55% and 66%, and BMPR-IB knock-down caused an approximate 94% increase in colony numbers compared with controls(Figure 3A, B).

### Effects of BMPR-IB overexpression and knock-down on the differentiation of glioblastoma cells in vitro

**Figure 4:**
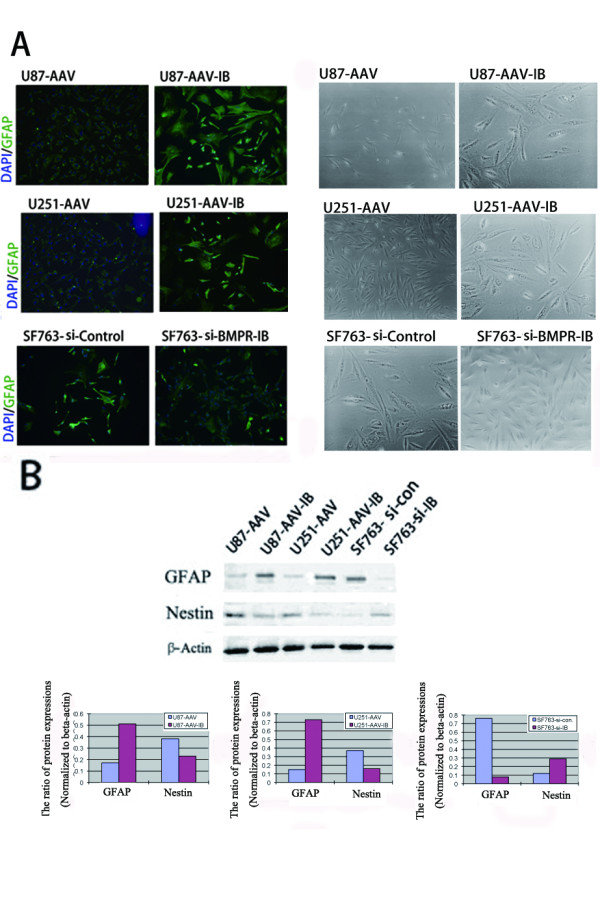
**Induction of differentiation by BMPR-IB in human glioma cell lines.** (**A**) After infection and transfection with rAAV-BMPR-IB and si-BMPR-IB, the expression of GFAP of glioblastoma cells was detected by immunofluorescence (left), and the morphological alterations were examined by phase contrast microscope(right). (**B**) WB analysis showed that BMPR-IB infection induced the expression of endogenous GFAP and inhibited the expression of Nestin, whereas BMPR-IB knock-down decreased the expression of GFAP and increased the expression of Nestin.

The contrast photomicrographs showed that the glioblastoma cell lines U87 and U251 were prone to differentiate after 2 days of rAAV-BMPR-IB infection. Conversely, BMPR-IB knock-down inhibited the outgrowth of neurites in SF763 cells (Figure 4A). Immunofluorescence analysis showed that BMPR-IB infection increased the expression of GFAP protein, which is a recognized marker of astrocytic differentiation, whereas BMPR-IB knock-down decreased the expression of GFAP protein (Figure 4A). Further investigation using western blot analysis showed that BMPR-IB overexpression increased the expression of GFAP protein and inhibited the expression of Nestin, which is a marker of CNS precursor cells. In addition, BMPR-IB knock-down decreased the expression of GFAP protein and increased the expression of Nestin protein (Figure 4B).

### Effects of altered BMPR-IB expression on the mRNA and protein levels of Skp2, p21, p27Kip1, Cdk2, Cdk4 and p53 in glioma cell lines

**Figure 5:**
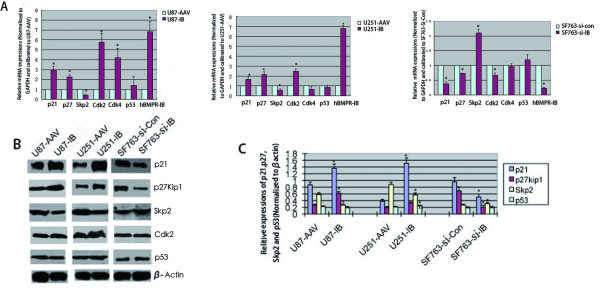
**Effects of altered BMPR-IB expression on the mRNA and protein expression of p21, CDK2, CDK4, p27Kip1, Skp2 and p53 in human glioma cell lines.** (**A**) Real-time RT-PCR was used to reveal alterations in the mRNA expression of p21, CDK2, CDK4, p27Kip1, Skp2 and p53 (values are expressed as the mean ± SD, n = 3. *, P < 0.05). (**B**) Western blot analysis showed alterations in the protein expression of p21, p27Kip1, Skp2 and p53 in these cell lines. Equal protein loading was monitored by hybridizing the same filter membrane with anti-beta-actin antibodies. (**C**) Statistical analysis of results from WB analysis. (Values are expressed as the mean ± SD, n = 3. *, P < 0.05).

To identify the mechanisms related to the growth inhibition and differentiation of glioma cells secondary to BMPR-IB overexpression, we first examined the mRNA expression of several cell cycle regulatory genes, including p21, p27Kip1, Skp2, Cdk2, Cdk4 and p53, by real-time RT-PCR and found increased expression of p21, p27, and Cdk2 mRNA, decreased expression of Skp2 mRNA, and consistent expression of p53 mRNA after 96 h of BMPR-IB infection of U87 and U251 cells (Figure 5A). We further examined whether BMPR-IB influences the protein expression of p21, p27Kip1, Skp2 and p53 by western blot analysis. We found a significant increase in the expression levels of the p21 and p27 proteins. The level of expression of the Skp2 protein, which is the specific recognition factor for p27Kip1 ubiquitination, was significantly lower in rAAV-BMPR-IB infected U87 and U251 cells compared with controls. Conversely, knock-down of BMPR-IB decreased the protein expression of p21 and p27 and increased the protein expression of Skp2. Additionally, Cdk2 and p53 proteins showed no significant changes in response to the alterations of the expression of BMPR-IB (Figure 5B).

### The effects of BMPR-IB overexpression and knock-down on the tumorigenicity of human glioblastoma cells in vivo

**Figure 6:**
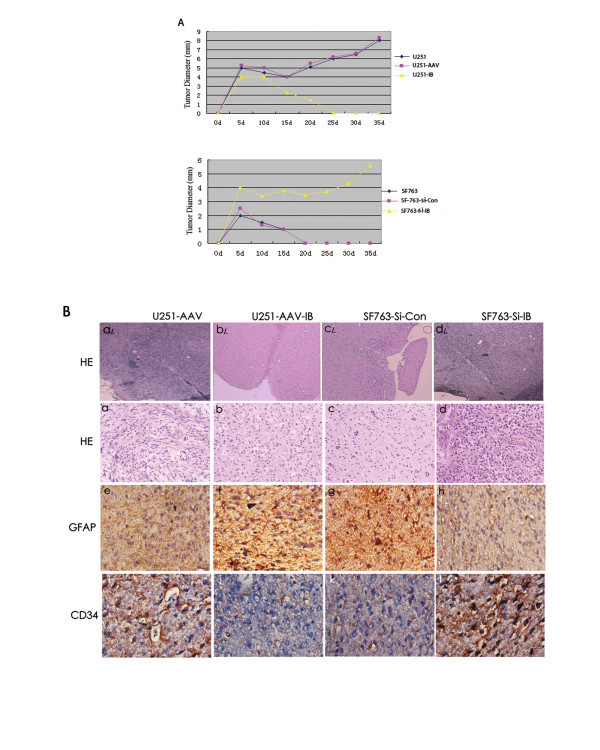
**Overexpression of BMPR-IB in human glioma cells decreased tumorigenicity in vivo.** (**A**) Tumor growth in the subcutis of nude mice. (**B**) Representative H&E staining and immunohistochemistry of tumors derived from intracranial xenografts of glioma cells.a_L_-d_L_(low magnification images)_L_ and a-d(high magnification images), HE staining of tumors derived from intracranial xenografts of glioma cells. e-h, GFAP immunohistocheistry of tumors derived from intracranial xenografts of glioma cells. i-l, CD34 immunohistocheistry of tumors derived from intracranial xenografts of glioma cells. (a, e, i, U251-AAV. b, f, j, U251-AAV-IB. c, g, k, SF763-si-control. d, h, l, SF763-si-IB). Magnification was ×20 in a-d, and ×40 in e-l. (**C**) Survival of animals intracranially injected with glioma cells that were infected or knocked down using BMPR-IB and control vectors (log rank test: p < 0.0001).

Additionally, we studied the kinetics of glioma cell growth using a subcutaneous xenograft and an intracranial xenograft in the nude mouse model system. As shown in Figure 6A, primary U251 cells and control vector-rAAV infected U251 (U251-AAV) cells (3× 10^6^ per mouse) formed aggressive, rapidly growing tumors that reached a diameter of ≥ 8 mm within 40 days after tumor cell injection. In contrast, U251-AAV-IB cells (3×10^6^ per mouse) formed tiny masses (≤ 4 mm in diameter) in nude mice by day 5 after injection. However, these masses shrank and disappeared within 25 days. The masses did not grow back over the following 4 weeks (Additional file 1: Figure S 3); thus, the formation of these masses could have been the result of an inflammatory reaction to the tumor cell injections. Conversely, inhibition of BMPR-IB caused malignant SF763 glioma cells to exhibit increased growth and regain tumorigenicity in the nude mouse model system (Figure 6A, Additional file 1: Figure S 3).

Next, to study the growth of these glioma cells in the brain, we used a xenograft model of human glioma, in which we injected glioma cells intracranially into nude mice. As with the subcutaneously injected cells, intracranially injected U251-AAV cells (1×10^7^ per mouse) formed invasive brain tumors that presented characteristic glioblastoma features, including nuclear pleomorphism, prominent mitotic activity, and highly invasive behavior (Figure 6B). These tumor masses also exhibited microvascular proliferation characterized by a substantially increased number of CD34-positive microvessels (Additional file 1: Figure S 4). Intracranial injection of U251-AAV-IB cells (1× 10^7^ per mouse) did not result in the formation of invasive tumors; instead, small, delimited lesions confined to the injection site were observed 90 days after injection. Immunohistology showed that these tumor masses presented a more mature morphology than that in control groups, characterized by the increased expression of GFAP, and less ventricular invasion. Furthermore, Kaplan–Meier survival analysis showed that BMPR-IB overexpression significantly extended the survival time of the mice compared with the controls (P < 0.0001; Figure 6B, C). Conversely, SF763 si-control infected cells did not produce tumors intracalvarially in injected mice; however, the SF763-Si-BMPR-IB cells produced invasive brain tumors intracalvarially, which resulted in decreased overall survival time compared with controls (P < 0.0001, Figure 6B, C).

## Discussion

Although several studies have suggested that BMPR-IB plays an important role in the development of some solid tumors, such as prostate cancer and breast cancer [14,15], its role and associated molecular mechanisms related to the development of glioma are not completely understood. In our study, we found both clinical and experimental evidence that aberrant BMPR-IB expression critically regulates the tumorigenicity of human glioma cells in vitro and in vivo [5]. We also provided the first evidence that BMPR-IB inhibits the growth of glioblastoma cells and promotes their differentiation by regulating the gene expression of p21, p27Kip1 and Skp2, which plays important roles in cell cycle regulation.

Previously, in clinical glioma specimens, we found decreased expression of BMPR-IB mRNA and protein in malignant glioma tissues compared to the levels in normal brain tissues and benign glioma tissues, whereas the expression of other molecules in the signaling pathway of BMPs/Smad1/5/8 remained consistent. We also found an inverse correlation between the protein and mRNA expression levels of BMPR-IB and malignancy grade [5]. From these clinical results, we assumed that BMPR-IB must be involved in the development of glioma. So, in our present study, we selected several malignant human glioblastoma cell lines that have different expressions of BMPR-IB to study the functional role of BMPR-IB in the development of glioma. Because the malignant human glioma cell lines that we selected have different expression levels of BMPR-IB, they are suitable as subjects for the study of the functional roles of BMPR-IB in vitro.

Hyperproliferation is a hallmark of glioblastoma multiforme. Our present study showed that BMPR-IB overexpression decreased the anchorage-independent growth of U87 and U251 glioblastoma cells, which present a lower expression of BMPR-IB in vitro. Further, the reduced BMPR-IB expression caused an increase in the number of SF763 colonies that express higher levels of BMPR-IB compared to other glioma cell lines. Additionally, FACS analysis showed that this effect was at least partially caused by the inhibition of glioma cell cycle progression at the G0/G1 transition (Figure 2B3B). These data suggest that BMPR-IB protein plays an inhibitory role in the development of glioblastoma and might be a key regulator of the G1-S transition in glioblastoma cells. A recent study by Piccirillo et al. has also shown that BMP4 may act as a key inhibitory regulator of tumor-initiating, stem-like CD133+ cells from GBMs. However, those authors did not address the aberrant expression of BMPR-IB in the primary tumor-initiating cells that were derived from GBM tissues [16]. We detected the expression of CD133 in U251/U87/SF763 cell lines, and found that most of these cells were CD133- (Additional file 1: Figure S 2). So, the tumor inhibited effects of BMPR-IB in our study are on those glioblstoma cells that express a low level of BMPR-IB, but are not limited to the fraction of cells with a stem cell-like phenotype (CD133+ cells) as reported by Piccirllo. et al.

It has been reported that BMP2/4 acts as a neuroepithelial proliferation signal at very early stages of embryonic central nervous system development, an effect mediated principally by BMPR1A [17,18]. Later in the development of the central nervous system, BMP2/4 induces neuronal and astrocytic differentiation of NSCs, an event that coincides with increased expression of BMPR1B [19,20]. Another study by Lee et al. has shown that BMPR-IB was able to induce the differentiation of a kind of gliomblastoma initiated cell [21]. In accordance with their findings, our present results suggest that human glioblastoma cell lines retain the BMPR-IB-mediated, signaling pathway that is responsible for growth arrest and astrocytic differentiation. Thus, our studies, together with their findings, should provide an attractive therapeutic strategy for the treatment of glioblastoma.

Although, Lee et al. also reported that BMPR-IB could induce the differentiation of a kind of gliomblastoma initiated cell, they did not clarify the signaling pathway that mediated these effects [21]. In our previous study, we found that transient overexpression of BMPR-IB could induce the phosphorylation and nuclear translocation of Smad1/5/8, which is the signaling molecule immediately downstream from BMPR-IB [5]. However, the detailed mechanism underlying the involvement of BMPR-IB in the growth inhibition and differentiation of glioblastoma remain indistinct. In the present study, we provide the first evidence to show that the selective induction of the key Cdk inhibitors (p27 Kip1 and p21) is associated with this growth arrest and differentiation processes.

The p27Kip1 is a potent tumor suppressor gene and an inhibitor of the cell cycle [22]. P27Kip1 plays its suppressive role through kinase-cyclin complexes that inhibit the phosphorylation of Rb that, in turn, results in the arrest of cells at the G1 phase. Deregulated expression of p27Kip1 plays a critical role in the pathogenesis of many human tumors. However, mutations of the p27Kip1 gene seem to be extremely rare in human malignancies [23]. Several studies have shown that nuclear expression of p27Kip1 decreases with malignancy in human astrocytic gliomas and that p27Kip1 has an independent prognostic value in patients who have malignant glioma [24,25]. Recently, Skp2 was shown to mediate the ubiquitin-mediated degradation of p27Kip1 as a specific substrate-recognition subunit and to have oncogenic properties [26]. The study of Schiffer et al. showed that the Skp2 expression level is directly correlated with glioma grade, but inversely correlated with the p27Kip1 level [27]. In this study, we also observed that BMPR-IB overexpression up regulated the mRNA and protein expressions of p21 and p27Kip1and decreased the mRNA and protein expressions of Skp2. The protein expression of p53, which is important in cell cycle progression and apoptosis in tumors, remained constant in these glioblastoma cell lines, regardless of BMPR-IB infection (Figure 5). Thus, the molecular mechanisms by which BMPR-IB induces the growth arrest and differentiation of glioma cells are associated with upregulation of the cell cycle kinase inhibitors p21 and p27Kip1, but not p53. Finally, p27Kip1 has been shown to modulate apoptosis in various types of cells, including glioblastoma multiforme cells [28,29]. In addition, in our previous study [5], we also observed early apoptosis in the glioblastoma cells, after transient transfection of BMPR-IB for 48 h. Because apoptosis is a large and important problem and thus we would continue to study the apoptotic effect and the mechanisms in the future clearly and in detail.

Based on these findings, we inferred that the growth arrest and differentiation of glioblastoma cells induced by BMPR-IB overexpression in vitro might correspond to a similar decline in the ability of rAAV-BMPR-IB infected cells to form tumors in vivo. This supposition was validated by our nude models of glioblastoma xenografts. All animals that received U251-AAV cells developed subcutaneous and intracranial tumor masses (Figure 6A, B). These masses showed characteristic glioblastoma features, including atypical nuclei, expression of aberrant glia and extensive neovascularization (Figure 6B). Conversely, U251-AAV-IB cells did not form invasive tumors(Figure 6A, B). Instead, rather, small, delimited lesions were observed, which were confined to the injection site. These tumors exhibited a more mature morphology (Figure 6B). Kaplan–Meier survival analysis showed that, after three to four months of post-intracalvarial injection, most of the control animals died, whereas nearly all of the mice that received rAAV-BMPR-IB infected cells survived (Figure 6C). Furthermore, BMPR-IB siRNA transfected SF763 cells showed reduced expression of BMPR-IB and regained tumorigenicity in most of the injected mice (Figure 6A, B, C). Thus, these results imply that BMPR-IB may play a role in glioma progression in vitro and in vivo.

In summary, our results show that overexpression of BMPR-IB clearly inhibited the growth, and promoted the differentiation, of glioma cells in vitro. In an animal model system, overexpression of BMPR-IB significantly inhibited the tumorigenicity of glioblastoma cells, whereas reduced expression of BMPR-IB significantly enhanced the tumorigenicity of these glioblastoma cells. Importantly, overexpression of BMPR-IB activated the BMPs/Smad1/5/8 signaling pathway and clearly inhibited the growth of glioma cells through multiple mechanisms, including decreased expression of Skp2, and subsequently increased the expression of the p21 and p27Kip1 proteins. Our results imply that BMPR-IB may play an inhibitory role in glioma progression, and that targeting BMPR-IB could represent a novel therapeutic approach to control malignant gliomas.

## Grant support

Chinese National Science Foundation:81172384

Chinese National Science Foundation:30873029

Chinese National Key Basic Research Project: 2009CB529400.

## Competing interests

The authors declare that they have no competing interests.

## Authors’ contributions

SL carried out the design of the experiments, performed most of experiments and drafted the manuscript. FY participated in establishing the nude models of glioblastoma. SWW and XRG participated in the experiments of cell culture and molecular biology. WHF participated in statistical analysis and interpretation. ZMT, JNZ and MF participated in the design of the experiments. All authors read and approved the final manuscript.

## Supplementary Material

Additional file 1 **Figure S1** The efficiency of AAV infection to U251 and U87 cells. U251 and U87 cells were infected with AAV vectors for 48 h, and then photographed using fluorescence microscope. **Figure S2** The expression of CD133 in glioblastoma cell lines and brain tumor stem cells (BTSCs). Immunofluorescence was used to detect the expression of CD133 in U251, U87, and SF763 glioblastoma cell lines and the neurospheres of BTSCs. **Figure S3** BMPR-IB inhibited the subcutaneous growth of glioblastoma cells. A) The subcutaneous models of nude glioblastoma cells, which over-expressed of BMPR-IB and knocked down BMPR-IB. B) The tumor masses derived from the subcutaneous xenograft. C) H&E staining of tumors derived from subcutaneous xenografts of glioblastoma cells. N: Normal connective tissue; T: Glioblastoma tissue. **Figure S4** Quantitative analysis of CD34 positive microvessels in the glioblastoma specimens. Glioblastoma specimens that were derived from U251-C/U251-IB and SF763-si-Con/SF763-si-IB cells were stained by CD34 using immunohistochemistry method. Error bars represent SD (performed in triplicate). *p < 0.01. **Table S1** Primer sequences for p21, p27, p53, CDK2, CDK4, Skp2, BMPR-IB (human) and GAPDH. Click here for file
