# Supplementary material for: Over-expression of BMPR-IB reduces the malignancy of glioblastoma cells by upregulation of p21 and p27Kip1
Source: J Exp Clin Cancer Res. 2012 May 31;31(1):52. doi: 10.1186/1756-9966-31-52 (PMC3408360; doi:10.1186/1756-9966-31-52)
Supplement: Additional file 1 — Figure S1 The efficiency of AAV infection to U251 and U87 cells. U251 and U87 cells were infected with AAV vectors for 48 h, and then photographed using fluorescence microscope. Figure S2 The expression of CD133 in glioblastoma cell lines and brain tumor stem cells (BTSCs). Immunofluorescence was used to detect the expression of CD133 in U251, U87, and SF763 glioblastoma cell lines and the neurospheres of BTSCs. Figure S3 BMPR-IB inhibited the subcutaneous growth of glioblastoma cells. A) The subcutaneous models of nude glioblastoma cells, which over-expressed of BMPR-IB and knocked down BMPR-IB. B) The tumor masses derived from the subcutaneous xenograft. C) H&E staining of tumors derived from subcutaneous xenografts of glioblastoma cells. N: Normal connective tissue; T: Glioblastoma tissue. Figure S4 Quantitative analysis of CD34 positive microvessels in the glioblastoma specimens. Glioblastoma specimens that were derived from U251-C/U251-IB and SF763-si-Con/SF763-si-IB cells were stained by CD34 using immunohistochemistry method. Error bars represent SD (performed in triplicate). *p < 0.01. Table S1 Primer sequences for p21, p27, p53, CDK2, CDK4, Skp2, BMPR-IB (human) and GAPDH. [file 1756-9966-31-52-S1.doc]

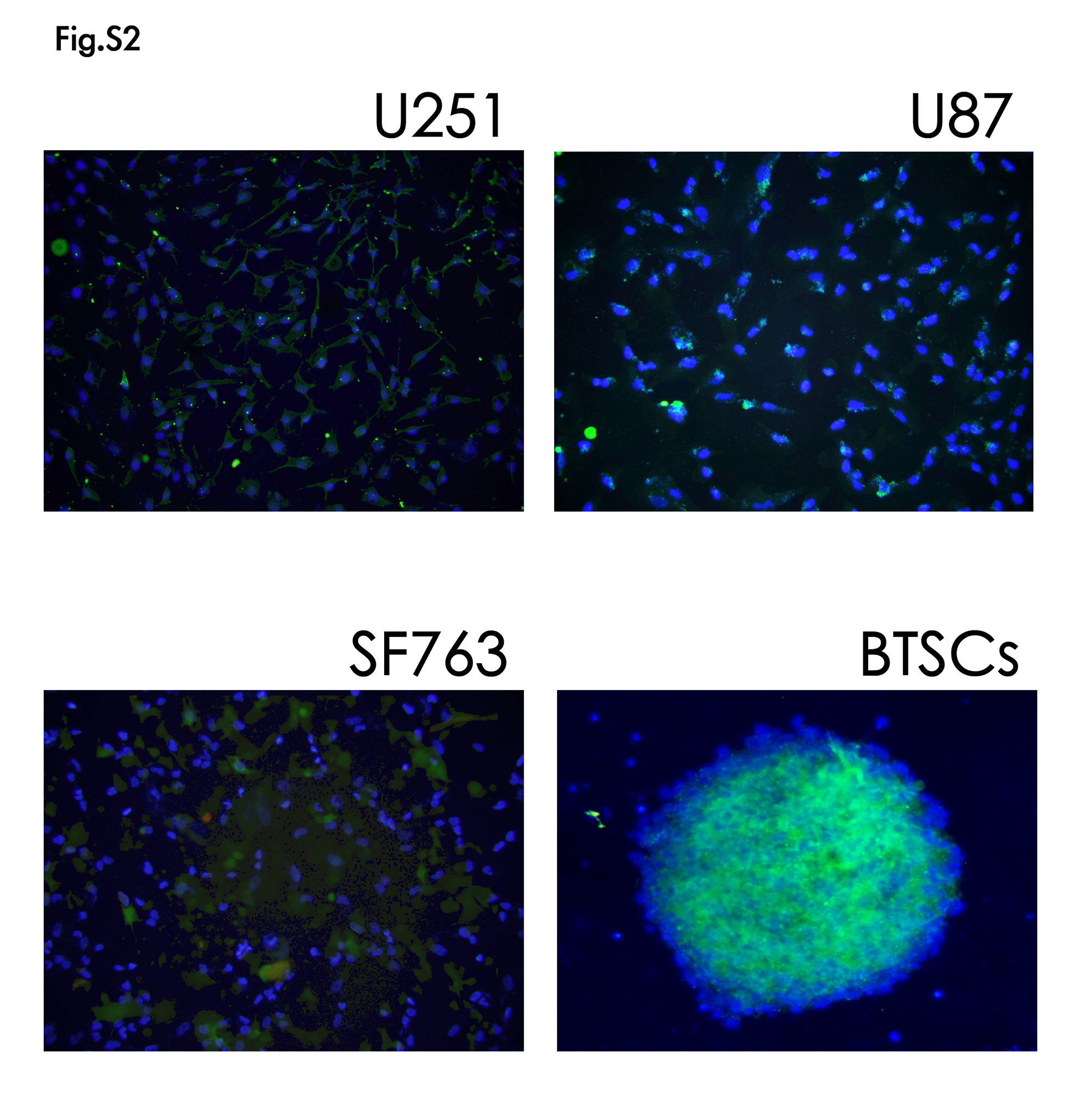


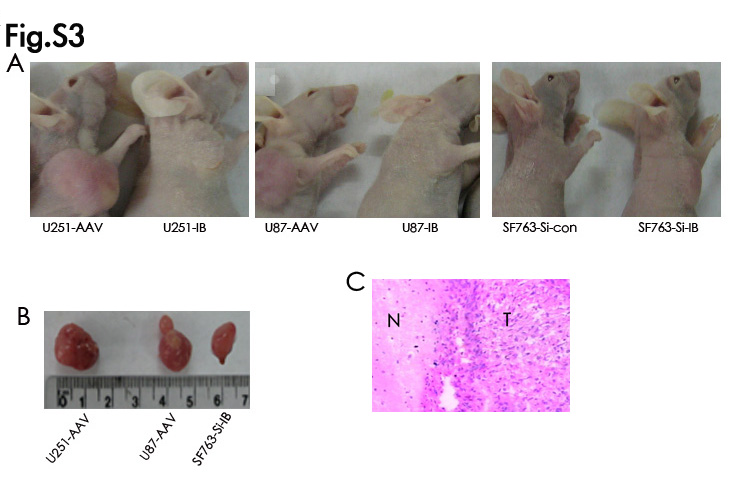


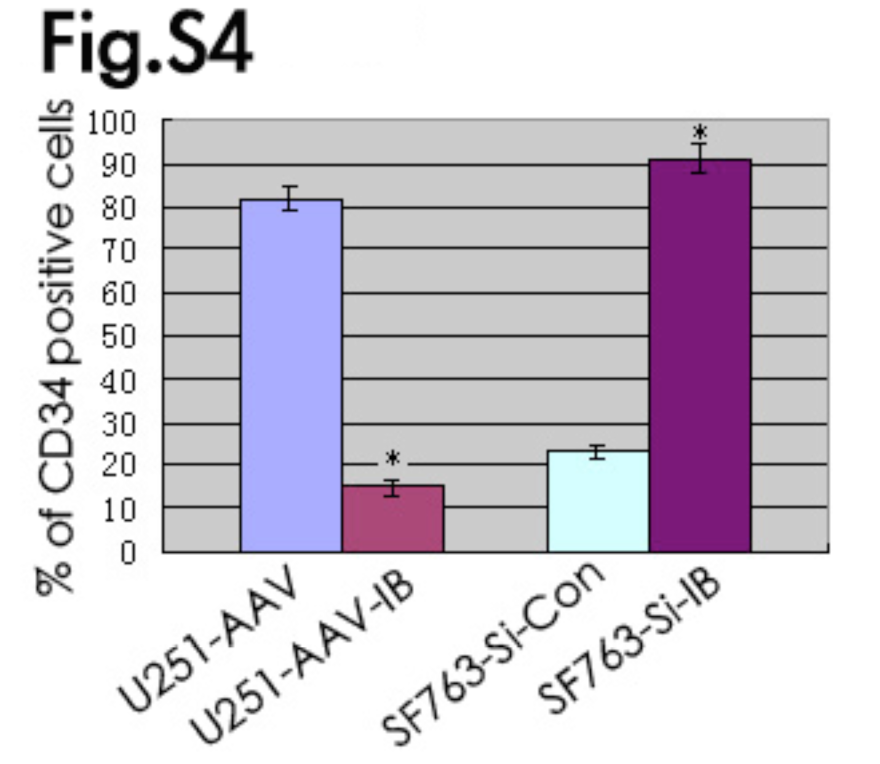


**Table S1**. The primer sequences of p21, p27, p53, CDK2, CDK4, Skp2, BMPR-IB (human) and GAPDH.

| **Genes** | **forward primer** | **reverse primer** |
| --- | --- | --- |
| P21 | 5' -AAGGTTGGCTCGTTCTCG -3' | 5'- GCTCATCTGCTGCCTGTAT -3' |
| P27 | 5’-GAGATTGTGGAGTTCGGCTCT-3’ | 5’-AGACAGAAAGTAGGATGGTGGG-3’ |
| P53 | 5'-CCACCATCCACTACAACTACAT-3' | 5'- AGGACAGGCACAAACACG -3' |
| Skp2 | 5'- ATGTGACTGGTCGGTTGC- 3' | 5' -GGAGGGTGGACACTTCTAT -3' |
| Cdk2 | 5'- CATTCTCATCGGGTCCTC- 3' | 5'- AAGCTCTGGCTAGTCCAA- 3' |
| Cdk4 | 5' -CGTGAGGTGGCTTTACTG -3' | 5'- TGTCCTTAGGTCCTGGTCT -3' |
| BMPR-IB | 5' –CCCCTCATTCCCAAACCG- 3' | 5' –TTAACCCGCAGGGCTGTC- 3' |
| GAPDH | 5’-GAAGGTGAAGGTCGGAGTC-3’ | 5’-GAAGATGGTGATGGGATTTC-3’ |
